# Protecting Chinese healthcare workers while combating the 2019 novel coronavirus

**DOI:** 10.1017/ice.2020.60

**Published:** 2020-03-05

**Authors:** Pengcheng Zhou, Zebing Huang, Yinzong Xiao, Xun Huang, Xue-Gong Fan

**Affiliations:** 1 Infection Control Centre, Xiangya Hospital, Central South University, Changsha, China; 2 Hunan Key Laboratory of Viral Hepatitis & Department of Infectious Diseases, Xiangya Hospital, Central South University, Changsha, China; 3 Burnet Institute, St Vincentʼs Hospital Melbourne, and University of Melbourne, Melbourne, Australia

*To the Editor*—Hospital-associated transmission is an important route of spreading the 2019 novel coronavirus SARS-CoV-2 and pneumonia (coronavirus disease 2019, COVID-19).^1^ Healthcare workers (HCWs) are at high risk while combating COVID-19 at the very front line, and nosocomial outbreaks among HCWs are not unusual in similar settings. The 2003 severe acute respiratory syndrome (SARS) outbreak led to >966 HCW infections with 1.4% deaths in mainland China.^2^ As of February 11, 2020, 3,019 HCWs might have been infected with SARS-CoV-2 in China, and 1,716 HCW cases of COVID-19 have been confirmed by nucleic acid testing^.3^ At least 6 HCWs have died, including the famous whistleblower Dr Li Wenliang. In view of this severe situation, we are recommending urgent interventions to help to protect HCWs.

A few aspects of COVID-19 have created a more severe situation than expected among HCWs. First, many infected individuals present with a typical symptoms, such as gastrointestinal symptoms and fatigue, or are asymptomatic.^4^ This situation may lead to a lack of recognition of the infection while patients are highly contagious. Furthermore, HCWs have not been not well prepared for this sudden COVID-19 outbreak, especially in departments other than infectious diseases. In Wuhan at the beginning of the outbreak, there was a general lack of awareness among HCWs to take precautions, and inadequate training among HCWs was noted, with staff incorrectly wearing personal protective equipment (PPE). In fact, ~30 HCWs in the Wuhan Mental Health Hospital were reported to be infected.^5^ Third, no point-of-care diagnostic assay was available in hospitals before late January 2019. In addition, the positive rate of the SARS-CoV-2 nucleic acid test kit remains relatively low even at present, and many patients have been diagnosed after >4 tests. These factors led to a diagnostic delay and opportunities for exposure among HCWs. Fourth, a good many tertiary and secondary hospitals are experiencing shortages of PPE and are calling for donations. HCWs have to use daily plastic products (photographic film, plastic wrap, file bag, and so forth) to make simple PPE. Lastly, some COVID-19 patients were admitted to the other departments by concealing their epidemiological history, which led to unnecessary exposure of HCWs.

Much can be done! We hope all countries and all people in the world can support the brave men and women on the front line of combating SARS-CoV-2. More PPE should be produced or imported, and it should be delivered to hospitals quickly. Training of HCWs to identify suspicious cases and to use PPE properly is urgently needed, especially for HCWs in departments other than infectious diseases. Furthermore, concealing medical history should have legal consequences.
